# Supplementary material for: Native biodiversity collapse in the eastern Mediterranean
Source: Proc Biol Sci. 2020 Dec 30;288(1942):20202469. doi: 10.1098/rspb.2020.2469 (PMC7892420; doi:10.1098/rspb.2020.2469)
Supplement: details on methods and additional results/figures and tables;Live-dead quantitative data;Mollusc sizes;Radiocarbon ages;R script [file rspb20202469supp1.docx]

**Native biodiversity collapse in the Eastern Mediterranean**

Supplementary material: details on methods and additional results/figures and tables

Paolo G. Albano^1^, Jan Steger^1^, Marija Bošnjak^1,2^, Beata Dunne^1^, Zara Guifarro^1^, Elina Turapova^1^, Quan Hua^3^, Darrell S. Kaufman^4^, Gil Rilov^5^, Martin Zuschin^1^

^1^ Department of Paleontology, University of Vienna, Althanstrasse 14, 1090 Vienna, Austria

^2^ Croatian Natural History Museum, Demetrova 1, Zagreb, Croatia

^3^ Australian Nuclear Science and Technology Organisation, Kirrawee DC, NSW 2232, Australia

^4^ School of Earth and Sustainability, Northern Arizona University, Flagstaff, Arizona 86011 USA

^5^ National Institute of Oceanography, Israel Oceanographic and Limnological Research (IOLR), Haifa 3108001, Israel

# Additional information on the methodology

## Study area and sampling sites

Table S1. List of sampling stations on the Mediterranean coast of Israel.

| **Station** | **Locality** | **Latitude [N]** | **Longitude [E]** | **Depth [m]** | **Date** | **Device** | **Substrate** | **Replicates** |
| --- | --- | --- | --- | --- | --- | --- | --- | --- |
| **Intertidal rocky substrate** | | | | | | | | |
| S8 | Tel Aviv | 32.08393 | 34.76573 | Intertidal | 27/04/2018 | Scraping | Breakwaters | 3 |
| S9 | Netanya | 32.32739 | 34.84591 | Intertidal | 29/04/2018 | Scraping | Breakwaters | 4 |
| S10 | Ashqelon | 31.68542 | 34.55967 | Intertidal | 30/04/2018 | Scraping | Breakwaters | 4 |
| S57 | Ashqelon | 31.68542 | 34.55967 | Intertidal | 31/10/2018 | Scraping | Breakwaters | 3 |
| S61 | Netanya | 32.32739 | 34.84591 | Intertidal | 02/11/2018 | Scraping | Breakwaters | 3 |
| S62 | Nahariyya | 33.01262 | 35.08973 | Intertidal | 06/11/2018 | Scraping | Rocky platform | 3 |
| S63 | Tel Aviv | 32.08393 | 34.76573 | Intertidal | 08/11/2018 | Scraping | Breakwaters | 3 |
| **Subtidal soft substrate** | | | | | | | | |
| SG10 | off Ashqelon | 31.69530 | 34.55880 | 11 | 19/09/2016 | Grab | Sand | 5 |
| SG10 | off Ashqelon | 31.69499 | 34.55892 | 11 | 27/04/2016 | Grab | Sand | 3 |
| SG20 | off Ashqelon | 31.70020 | 34.54980 | 21 | 18/09/2016 | Grab | Sand | 5 |
| SG20 | off Ashqelon | 31.70097 | 34.55007 | 21 | 27/04/2017 | Grab | Sand | 3 |
| SG30 | off Ashqelon | 31.71000 | 34.54060 | 30 | 18/09/2016 | Grab | Sand | 5 |
| SG30 | off Ashqelon | 31.71036 | 34.54245 | 30 | 27/04/2017 | Grab | Sand | 3 |
| SG40 | off Ashqelon | 31.74870 | 34.49600 | 41 | 18/09/2016 | Grab | Sandy mud | 5 |
| SG40 | off Ashqelon | 31.74918 | 34.49636 | 41 | 27/04/2017 | Grab | Sandy mud | 3 |
| NG10 | off Atlit | 32.78200 | 34.94660 | 10 | 21/09/2016 | Grab | sand | 5 |
| NG10 | off Atlit | 32.78104 | 34.94556 | 11 | 25/04/2017 | Grab | sand | 3 |
| NG30 | off Atlit | 32.74220 | 34.91810 | 30 | 20/09/2016 | Grab | sand | 5 |
| NG30 | off Atlit | 32.74169 | 34.91773 | 30 | 25/04/2017 | Grab | Sand | 3 |
| **Subtidal rocky substrate** | | | | | | | | |
| S12 | off Ashqelon | 31.68683 | 34.55156 | 12 | 30/04/2018 | Suction sampler | Rocks | 3 |
| S13 | west of Rosh HaNikra Islands | 33.07247 | 35.09228 | 20 | 01/05/2018 | Suction sampler | Rocks | 3 |
| S14 | west of Rosh HaNikra Islands | 33.07037 | 35.09260 | 12 | 01/05/2018 | Suction sampler | Rocks | 3 |
| S16 | off Ashqelon | 31.68909 | 34.52569 | 25 | 02/05/2018 | Suction sampler | Rocks | 2 |
| S52 | west of Rosh HaNikra Islands | 33.07037 | 35.09260 | 12 | 29/10/2018 | Suction sampler | Rocks | 3 |
| S53 | west of Rosh HaNikra Islands | 33.07247 | 35.09228 | 20 | 29/10/2018 | Suction sampler | Rocks | 3 |
| S58 | off Ashqelon | 31.68683 | 34.55156 | 12 | 31/10/2018 | Suction sampler | Rocks | 3 |
| S59 | off Ashqelon | 31.68909 | 34.52569 | 28 | 31/10/2018 | Suction sampler | Rocks | 3 |
| **Mesophotic rocky substrate** | | | | | | | | |
| CH2 | Rosh Carmel | 32.87793 | 34.86118 | 92 | 25/06/2018 | Grab | Rocks | 1 |
| RC6 | Rosh Carmel | 32.87793 | 34.86118 | 92 | 25/06/2018 | Grab | Rocks | 1 |
| RC16 | Rosh Carmel | 32.87793 | 34.86118 | 92 | 25/06/2018 | Grab | Rocks | 1 |
| RC27 | Rosh Carmel | 32.87793 | 34.86118 | 92 | 25/06/2018 | Grab | Rocks | 1 |
| RC33 | Rosh Carmel | 32.87793 | 34.86118 | 92 | 25/06/2018 | Grab | Rocks | 1 |
| RC36 | Rosh Carmel | 32.87793 | 34.86118 | 92 | 25/06/2018 | Grab | Rocks | 1 |
| RC44 | Rosh Carmel | 32.87793 | 34.86118 | 92 | 25/06/2018 | Grab | Rocks | 1 |
| RC50 | Rosh Carmel | 32.87793 | 34.86118 | 92 | 25/06/2018 | Grab | Rocks | 1 |
| RC51 | Rosh Carmel | 32.87793 | 34.86118 | 92 | 25/06/2018 | Grab | Rocks | 1 |
| RC60 | Rosh Carmel | 32.87793 | 34.86118 | 92 | 25/06/2018 | Grab | Rocks | 1 |
| RC61 | Rosh Carmel | 32.87793 | 34.86118 | 92 | 25/06/2018 | Grab | Rocks | 1 |
| RC66 | Rosh Carmel | 32.87793 | 34.86118 | 92 | 25/06/2018 | Grab | Rocks | 1 |
| **Mesophotic soft substrate** | | | | | | | | |
| TG80 | Off Atlit | 32.80770 | 34.85371 | 77-83 | 21/09/2016 | Grab | Mud | 3 |


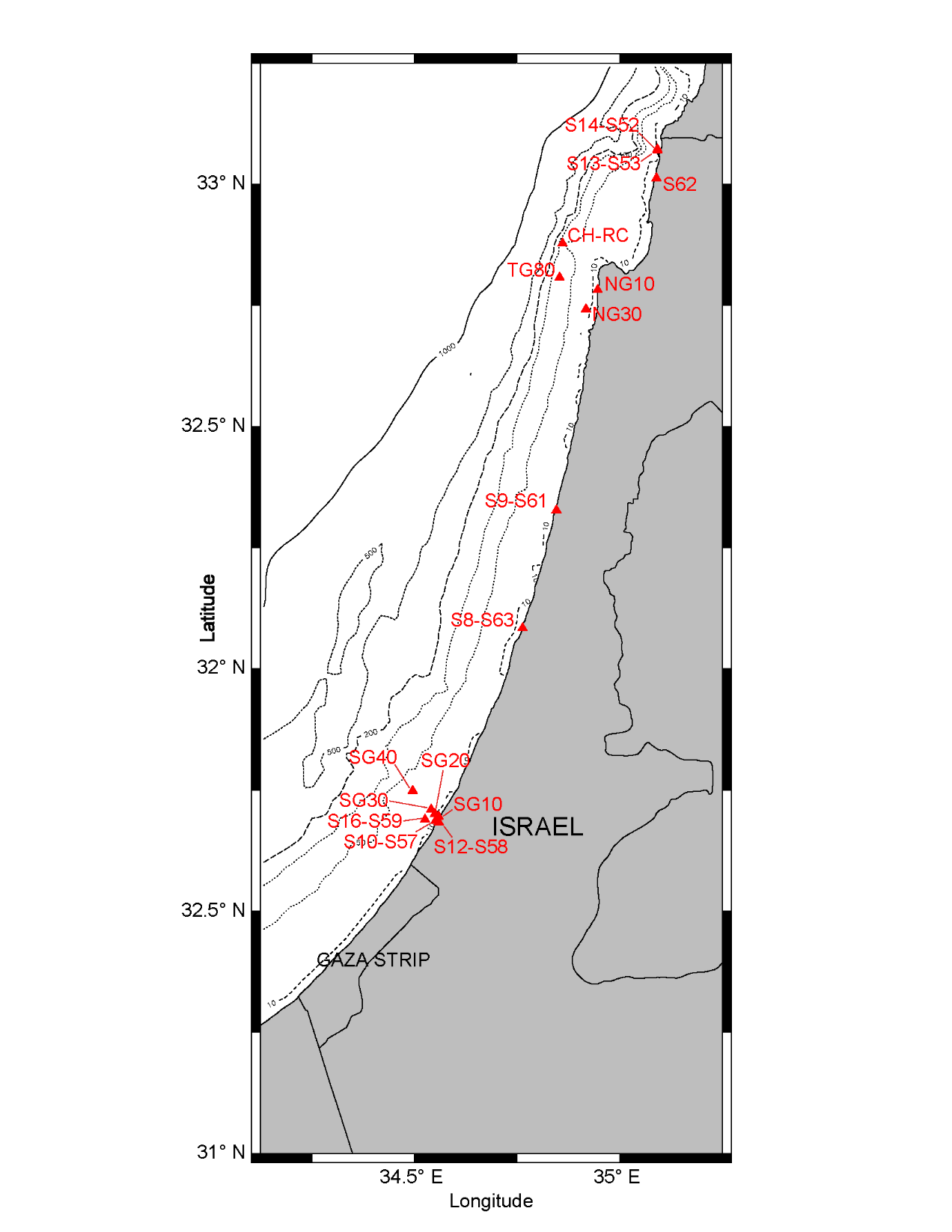


Figure S1. Map of sampling stations on the Mediterranean coast of Israel.

## Checklist for the intertidal samples

We reviewed the available literature on Israeli marine mollusks, in particular (Barash & Danin, 1992), and selected as pool for comparison the species potentially occurring on upper intertidal rocky substrates similar to the ones we sampled. The Israeli shores north of Haifa Bay present more frequent intertidal rocky shores than the southern ones, which are mostly sandy (and nowadays host the artificial breakwaters we sampled). This is reflected in different species pools for the southern sites (Ashqelon, Tel Aviv, Netanya) and the northern one (Nahariyya). Barash & Danin (1992) reported also *Patella rustica* Linnaeus, 1758, but this record is likely erroneous (H. Mienis, pers. comm., 7 November 2019).

Table S2. Checklist of mollusks of the surveyed rocky intertidal Mediterranean Israeli coastline.

| **Class** | **Family** | **Genus** | **Species** | **Author** | **Status** | **South** | **North** |
| --- | --- | --- | --- | --- | --- | --- | --- |
| Gastropoda | Patellidae | *Patella* | *caerulea* | Linnaeus, 1758 | Native | X | X |
| Gastropoda | Patellidae | *Patella* | *ulyssiponensis* | Gmelin, 1791 | Native |  | X |
| Gastropoda | Nacellidae | *Cellana* | *rota* | (Gmelin, 1791) | NIS | X | X |
| Gastropoda | Trochidae | *Phorcus* | *articulatus* | (Lamarck, 1822) | Native |  | X |
| Gastropoda | Trochidae | *Phorcus* | *richardi* | (Payraudeau, 1826) | Native |  | X |
| Gastropoda | Trochidae | *Phorcus* | *turbinatus* | (Born, 1778) | Native | X | X |
| Gastropoda | Trochidae | *Steromphala* | *rarilineata* | (Michaud, 1829) | Native |  | X |
| Gastropoda | Littorinidae | *Echinolittorina* | *punctata* | (Gmelin, 1791) | Native | X | X |
| Gastropoda | Littorinidae | *Melaraphe* | *neritoides* | (Linnaeus, 1758) | Native | X | X |
| Gastropoda | Siphonariidae | *Siphonaria* | *crenata* | Blainville, 1827 | NIS | X | X |
| Bivalvia | Mytilidae | *Brachidontes* | *pharaonis* | (P. Fischer, 1870) | NIS | X | X |
| Bivalvia | Mytilidae | *Musculus* | *costulatus* | (Risso, 1826) | Native | X | X |
| Bivalvia | Mytilidae | *Mytilaster* | *minimus* | (Poli, 1795) | Native | X | X |

## Radiocarbon dating and calibration procedures

We dated 149 valves (Table S3) by accelerator mass spectrometry (AMS), using powdered carbonate targets (Bright et al., submitted; Bush et al., 2013), with a typical analytical precision of better than 0.6% (1σ).

Table S3. List of radiocarbon dated material.

| **Sample** | **Species** | **Number of valves** | **Year of collection** |
| --- | --- | --- | --- |
| SG10 | *Donax semistriatus* | 15 | 2016 |
| SG20 | *Corbula gibba* | 15 | 2016 |
| SG30 | *Corbula gibba* | 15 | 2016 |
| SG40 | *Corbula gibba* | 15 | 2016 |
| NG10 | *Striarca lactea* | 15 | 2016 |
| NG30 | *Corbula gibba* | 15 | 2016 |
| S12-S58 | *Striarca lactea* | 10 | 2018 |
| S13-S53 | *Striarca lactea* | 10 | 2018 |
| S14-S52 | *Striarca lactea* | 10 | 2018 |
| S16-S59 | *Striarca lactea* | 10 | 2018 |
| TG80 | *Corbula gibba* | 10 | 2016 |
| RC16 | *Striarca lactea* | 9 | 2018 |

To allow for a sufficient mass for radiocarbon analysis, we selected the shells with mass larger than 0.5 mg. Mollusk shells were subsampled by gently breaking and selecting a small fragment. All samples were cleaned by sonicating and rinsing in deionized distilled water (DDI; 16.3 Mohm·cm) repeatedly up to three times. Samples were leached with 2 M HCl, with the extent of leaching dependent on sample mass: samples larger than 1 mg were leached to remove about 30% by mass and samples between about 1 and 0.5 mg were leached to remove about 15%. Samples were ultimately rinsed three times with DDI water then dried in a 50 °C oven overnight. They were ground to a fine powder using a small agate mortar and pestle. Between 0.15 and 0.50 mg of the carbonate powder was transferred to serialized (3 hr at 500 °C) borosilicate glass culture tubes (6 mm OD x 50 mm). Samples comprising less than 0.15 mg of recovered powder were not analyzed. The carbonate was combined with 6 to 7 mg of niobium (Nb Puratronic, -325 mesh, 99.99%) powder using a spatula. The tubes were flushed with N_2_ gas and capped with Supelco plastic column caps (1/4” OD) to reduce atmospheric exposure until the powder was pressed into targets.

The metal plus the carbonate mixture was pressed into pre-drilled (0.160” depth) aluminum targets at 400 psi, rotated 90°, and pressed again at 400 psi. The targets were sent to the Keck Carbon Cycle AMS Laboratory at the University of California Irvine for ^14^C analysis.

Radiocarbon ages were converted to calendar years using OxCal 4.2 (Bronk Ramsey, 2009), Marine13 data (Reimer et al., 2013), and a constant regional marine reservoir correction (ΔR) of 3 ± 66 yrs, which is the weighted mean of eight published pre-bomb ΔR values from Israel and Lebanon (see Table S4). For samples younger than 1950 AD, the fraction of modern carbon (F^14^C) was converted to calendar ages using a regional marine calibration curve and the calibration software OxCal v4.2. The post-1950 regional marine curve was constructed using 10 live-collected *Corbula gibba* shells collected along the coast of Israel (see Table S5).

Table S4. Regional pre-bomb ΔR values for our study sites. These pre-bomb ΔR values, listed in the Online Marine Reservoir Correction Database (http://calib.org/marine/), were used for the calculation of a weighted mean ΔR value of 3 ± 66 ^14^C yr (n=8).

|  | **Location** | **Latitude** | **Longitude** | **Year of collection** | **ΔR ± 1σ (^14^C yr)** | **References** |
| --- | --- | --- | --- | --- | --- | --- |
| 1 | Netamiya, Israel | 34.83 | 32.17 | AD 1937 | 52 ± 40 | Reimer and McCormac, 2002 |
| 2 | Beirut, Lebanon | 35.5 | 33.87 | AD 1929 | 37 ± 40 | Reimer and McCormac, 2002 |
| 3 | Beirut, Lebanon | 35.5 | 33.87 | AD 1929 | -52 ± 50 | Reimer and McCormac, 2002 |
| 4 | Israel | 34.8482 | 32.3384 | AD 1937 | 47 ± 40 | Boaretto et al., 2010 |
| 5 | Israel | 34.8482 | 32.3384 | AD 1937 | -70 ± 50 | Boaretto et al., 2010 |
| 6 | Israel | 34.9227 | 32.6432 | AD 1937 | -20 ± 50 | Boaretto et al., 2010 |
| 7 | Israel | 34.9227 | 32.6432 | AD 1937 | 75 ± 50 | Boaretto et al., 2010 |
| 8 | Israel | 35.0138 | 32.8431 | AD 1937 | -115 ± 50 | Boaretto et al., 2010 |

Table S5. ^14^C values of live-collected shells (*Corbula gibba*) measured in this study. All the shell samples were collected at water depth of 50 m or less, and were converted to graphite and measured using the AMS Facility at the University of California Irvine. These measured ^14^C values were used to constructed the post-1950 regional marine curve for age calibration.

|  | **Lab ID** | **Sample ID** | **Location** | **Year of collection** | **F^14^C ± 1σ** |
| --- | --- | --- | --- | --- | --- |
| 1 | 214201 | RC198 | Israel, Haifa Bay | AD 1954 | 0.9468 ± 0.0020 |
| 2 | 214202 | RC200 | Israel, Ashdod | AD 1960 | 0.9860 ± 0.0018 |
| 3 | 214203 | RC202 | Israel, Bat Yam | AD 1962 | 0.9937 ± 0.0019 |
| 4 | 207180 | RC096 | Israel, Atlit-Dor | AD 1965 | 1.0670 ± 0.0020 |
| 5 | 207179 | RC098 | Israel, Ashdod | AD 1970 | 1.1241 ± 0.0018 |
| 6 | 207178 | RC100 | Israel, Palmachim | AD 1977 | 1.1177 ± 0.0020 |
| 7 | 207177 | RC102 | Israel, Nizzanim | AD 1988 | 1.0883 ± 0.0017 |
| 8 | 207176 | RC080 | Israel, Hadera | AD 2002 | 1.0499 ± 0.0017 |
| 9 | 207175 | RC092 | Israel, Atlit | AD 2016 | 1.0342 ± 0.0017 |
| 10 | 207174 | RC094 | Israel, Ashqelon | AD 2017 | 1.0293 ± 0.0017 |

## Determination of size at maturity

In the absence of a review on this topic, we recovered the size at maturity of 73 species of marine mollusks based on the work of Gosselin and Quin (1997) that listed age at maturity for numerous marine mollusks. We retrieved the size at maturity and the maximum size from the literature cited therein (Table S6). For species for which size intervals were reported, we used the mean of the extremes; when different size at maturity was recorded for the different sexes, we listed them as two independent records (also in order to take into account different maximum size between sexes).

The median ratio between size at maturity and maximum size is 0.56 and in 60% of the cases the ratio is larger than 0.5. Additionally, in only 22% of the cases this ratio is smaller than 0.4.

Figure S2. Frequency distribution of the ratio between size at maturity and maximum size of 73 species of marine mollusks. The blue line marks the median.

Table S6. Size at maturity for 73 species of marine mollusks compiled from the literature listed by Gosselin and Quin (1997).

| **Class** | **Family** | **Species** | **Size at maturity [mm]** | **Max size [mm]** | **Ratio** | **Source** | **Notes** |
| --- | --- | --- | --- | --- | --- | --- | --- |
| Polyplacophora | Lepidochitonidae | *Cianoplax fernaldi* (Eernisse, 1986) | NA | 15 | NA | NA | Was *Lepidochitona* in Gosselin & Qian 1997. Reference unavailable. Maximum size from (Eernisse, 1986) |
| Polyplacophora | Chitonidae | *Onithochiton*  *quercinus*  (Gould, 1846) | 23 | 80 | 0.29 | (Otway, 1994) |  |
| Polyplacophora | Mopaliidae | *Katharina*  *tunicata*  (Wood, 1815) | 33-36 | 55 | 0.60-0.65 | (Heath, 1905) | Not *Mopalia muscosa* as stated in Gosselin and Quin (1997) with ref. Heath (1907) |
| Polyplacophora | Mopaliidae | *Plaxiphora*  *albida*  (Blainville, 1825) | 24 | 80 | 0.30 | (Otway, 1994) |  |
| Polyplacophora | Acanthochitonidae | *Cryptochiton*  *stelleri*  (Middendorff, 1847) | 120-175 | 270 | 0.44-0.65 | (Heath, 1905) |  |
| Gastropoda | Lottiidae | *Discurria*  *insessa*  (Hinds, 1842) | 6 | 14.0 | 0.43 | (Choat & Black, 1979) | as *Acmaea* |
| Gastropoda | Lottiidae | *Lottia*  *digitalis*  (Rathke, 1833) | 11.5 | 26.0 | 0.44 | (Choat & Black, 1979) |  |
| Gastropoda | Nacellidae | *Cellana*  *tramoserica*  (Holten, 1802) | 12 | 32 | 0.38 | (Underwood, 1975) | presence of gonads, no spawning observed, might be underestimate of size at first reproduction |
| Gastropoda | Haliotidae | *Haliotis*  *australis*  Gmelin, 1791 | 60 | 80 | 0.75 | (Poore, 1973) |  |
| Gastropoda | Haliotidae | *Haliotis*  *cyclobates*  Péron & Lesueur, 1816 | 40 | 48 | 0.83 | (Shepherd, 1986) |  |
| Gastropoda | Haliotidae | *Haliotis*  *discus hannai*  Ino, 1953 | 70 | 90 | 0.78 | (Sakai, 1962) |  |
| Gastropoda | Haliotidae | *Haliotis*  *iris*  Gmelin, 1791 | 68 | 132 | 0.52 | (Poore, 1973) |  |
| Gastropoda | Haliotidae | *Haliotis*  *laevigata*  Donovan, 1808 | 95 | 120 | 0.79 | (Shepherd, 1986) |  |
| Gastropoda | Haliotidae | *Haliotis*  *roei*  Gray, 1826 | 43 | 57 | 0.75 | (Shepherd, 1986) |  |
| Gastropoda | Haliotidae | *Haliotis*  *rubra*  Leach, 1814 | 90 | 113 | 0.80 | (Shepherd, 1986) |  |
| Gastropoda | Haliotidae | *Haliotis*  *scalaris*  (Leach, 1814) | 60 | 70 | 0.86 | (Shepherd, 1986) |  |
| Gastropoda | Haliotidae | *Haliotis*  *tuberculata*  Linnaeus, 1758 | 50 | 90 | 0.56 | (Crofts, 1937) | Maximum size from (Fretter & Graham, 1976) |
| Gastropoda | Trochidae | *Umbonium*  *costatum*  (Kiener, 1839) | 11 | 22 | 0.50 | (Noda et al., 1995) | size as shell diameter |
| Gastropoda | Trochidae | *Umbonium*  *vestiarium*  (Linnaeus, 1758) | 7 | 12 | 0.58 | (Berry, 1989) | size as shell diameter |
| Gastropoda | Neritidae | *Nerita*  *atramentosa*  Reeve, 1855 | 13.5 | 21.5 | 0.63 | (Underwood, 1975) |  |
| Gastropoda | Capulidae | *Trichotropsis*  *cancellata*  Hinds, 1843 | 15-24 | 42 | 0.36-0.57 | (Yonge, 1962) |  |
| Gastropoda | Littorinidae | *Bembicium*  *vittatum*  Philippi, 1846 | 8.5 | 11.4 | 0.75 | (Robert Black et al., 1994) |  |
| Gastropoda | Littorinidae | *Bembicium*  *nanum*  (Lamarck, 1822) | 11 | 14.5 | 0.76 | (Underwood, 1975) | size as shell breadth |
| Gastropoda | Littorinidae | *Lacuna*  *pallidula*  (da Costa, 1778) | 7 | 8.4 | 0.83 | (Smith, 1973) |  |
| Gastropoda | Littorinidae | *Lacuna*  *vincta*  (Montagu, 1803) | 5 | 6 | 0.83 | (Smith, 1973) |  |
| Gastropoda | Littorinidae | *Littorina*  *littorea*  (Linnaeus, 1758) | 10-14 | 30 | 0.33-0.47 | (Hughes & Roberts, 1980) |  |
| Gastropoda | Littorinidae | *Littorina*  *saxatilis*  (Olivi, 1792) | 11-12 | 18 | 0.61-0.67 | (Hughes & Roberts, 1980) | as *L. nigrolineata*, females |
| Gastropoda | Littorinidae | *Littorina*  *saxatilis*  (Olivi, 1792) | 9-11 | 18 | 0.50-0.61 | (Hughes & Roberts, 1980) | as *L. nigrolineata*, males |
| Gastropoda | Littorinidae | *Littorina*  *saxatilis*  (Olivi, 1792) | 8-9 | 18.4 | 0.44-0.49 | (Hughes & Roberts, 1980) | as *L. rudis*, Aber population, females |
| Gastropoda | Littorinidae | *Littorina*  *saxatilis*  (Olivi, 1792) | 6-7 | 18.4 | 0.33-0.38 | (Hughes & Roberts, 1980) | as *L. rudis*, Aber population, males |
| Gastropoda | Littorinidae | *Littorina*  *saxatilis*  (Olivi, 1792) | 9-10 | 17 | 0.53-0.59 | (Hughes & Roberts, 1980) | as *L. rudis*, Trwyn-y-Penrhyn population, females |
| Gastropoda | Littorinidae | *Littorina*  *saxatilis*  (Olivi, 1792) | 7-8 | 17 | 0.41-0.47 | (Hughes & Roberts, 1980) | as *L. rudis*, Trwyn-y-Penrhyn population, males |
| Gastropoda | Littorinidae | *Littorina*  *saxatilis*  (Olivi, 1792) | 6-7 | 13 | 0.46-0.54 | (Hughes & Roberts, 1980) | as *L. rudis*, Llanddwyn population, females |
| Gastropoda | Littorinidae | *Littorina*  *saxatilis*  (Olivi, 1792) | 3-4 | 13 | 0.23-0.31 | (Hughes & Roberts, 1980) | as *L. rudis*, Llanddwyn population, males |
| Gastropoda | Littorinidae | *Littorina*  *sitkana*  Philippi, 1846 | 5.5-7.0 | 16 | 0.34-0.44 | (Boulding & Van Alstyne, 1993) | females |
| Gastropoda | Littorinidae | *Littorina*  *sitkana*  Philippi, 1846 | 4.2-6.0 | 16 | 0.26-0.38 | (Boulding & Van Alstyne, 1993) | males |
| Gastropoda | Littorinidae | *Littorina*  *subrotundata*  (Carpenter, 1864) | 3.8-5.5 | 6.43 | 0.59-0.86 | (Boulding & Van Alstyne, 1993) | females; as *Littorina* sp. (but see Reid, 1996); not max size, but mean adult size |
| Gastropoda | Littorinidae | *Littorina*  *subrotundata*  (Carpenter, 1864) | 3.3-5.0 | 6.43 | 0.51-0.78 | (Boulding & Van Alstyne, 1993) | males; as *Littorina* sp. (but see Reid, 1996); not max size, but mean adult size |
| Gastropoda | Littorinidae | *Melarhaphe*  *neritoides*  (Linnaeus, 1758) | 2.5-3.5 | 7.5 | 0.33-0.47 | (Hughes & Roberts, 1980) | as *Littorina* |
| Gastropoda | Strombidae | *Lobatus*  *gigas*  Linnaeus, 1758 | 240 | 340 | 0.71 | (Appeldoorn, 1990) |  |
| Gastropoda | Muricidae | *Drupella*  *cornus*  (Röding, 1798) | 28 | 42 | 0.67 | (R. Black & Johnson, 1994) |  |
| Gastropoda | Muricidae | *Nucella*  *canaliculata*  (Duclos, 1832) | 30 | 35 | 0.86 | (T. M. Spight, 1975) |  |
| Gastropoda | Muricidae | *Nucella*  *emarginata*  (Deshayes, 1839) | 17 | 26 | 0.65 | (Tom M. Spight, 1982) |  |
| Gastropoda | Muricidae | *Nucella*  *emarginata*  (Deshayes, 1839) | 21.5 | 30 | 0.72 | (T. M. Spight, 1975) |  |
| Gastropoda | Muricidae | *Nucella*  *lamellosa*  (Gmelin, 1791) | 29-35 | 60 | 0.41-0.50 | (T. M. Spight, 1975) |  |
| Gastropoda | Muricidae | *Nucella*  *lapillus*  (Linnaeus, 1758) | 16-20 | 20-35 | 0.57-0.80 | (Crothers, 1985) |  |
| Gastropoda | Buccinidae | *Buccinum*  *undatum*  Linnaeus, 1758 | 49-76 | 110 | 0.45-0.69 | (Gendron, 1992) | Males, maximum size from (Fretter & Graham, 1985) |
| Gastropoda | Buccinidae | *Buccinum*  *undatum*  Linnaeus, 1758 | 60-81 | 110 | 0.55-0.74 | (Gendron, 1992) | Females, maximum size from (Fretter & Graham, 1985) |
| Gastropoda | Buccinidae | *Busycon*  *carica*  (Gmelin, 1791) | 130 | 229 | 0.57 | (Castagna & Kraeuter, 1994) | Maximum size from (Abbott, 1974) |
| Gastropoda | Corambidae | *Corambe*  *steinbergae*  (Lance, 1962) | NA | NA | NA | NA | as *Doridella*, size not provided, only age (Strathmann, 1987) |
| Gastropoda | Tritoniidae | *Tritonia*  *tetraquetra*  (Pallas, 1788) | NA | 150 | NA | (Kemph & Willows, 1977) | as *T. diomedea* |
| Gastropoda | Eubranchidae | *Eubranchus*  *rustyus*  (Er. Marcus, 1961) | 6-10 | 8-15 (25) | 0.40-0.75 | (Robilliard, 1971) |  |
| Gastropoda | Trinchesiidae | *Phestilla*  *sibogae*  Bergh, 1905 | NA | NA | NA | NA | Size not provided, only age |
| Gastropoda | Trinchesiidae | *Trinchesia*  *alpha*  (Baba & Hamatani, 1963) | 6-7 | 5-13 | 0.46 | (Robilliard, 1971) | as *Catriona* |
| Gastropoda | Retusidae | *Retusa*  *obtusa*  (Montagu, 1803) | 3 | 5.5 | 0.55 | (Berry, 1989) |  |
| Gastropoda | Antarctophilinidae | *Antarctophiline*  *gibba*  (Strebel, 1908) | 9 | 12 | 0.75 | (Seager, 1982) | as *Philine* |
| Gastropoda | Aplysiidae | *Stylocheilus*  *longicauda*  (Quoy & Gaimard, 1825) | 1.85 | 15.5 | 0.12 | (Switzer-Dunlap & Hadfield, 1979) | in body mass [g] |
| Gastropoda | Aplysiidae | *Aplysia*  *juliana*  Quoy & Gaimard, 1832 | 25 | 210 | 0.12 | (Switzer-Dunlap & Hadfield, 1979) | in body mass [g] |
| Gastropoda | Aplysiidae | *Aplysia*  *dactylomela*  Rang, 1828 | 90 | 245 | 0.37 | (Switzer-Dunlap & Hadfield, 1979) | in body mass [g] |
| Gastropoda | Aplysiidae | *Dolabella*  *auricularia*  (Lightfoot, 1786) | 113 | 233 | 0.48 | (Switzer-Dunlap & Hadfield, 1979) | in body mass [g] |
| Gastropoda | Aplysiidae | *Dolabella*  *auricularia*  (Lightfoot, 1786) | 275 | 364 | 0.76 | (Switzer-Dunlap & Hadfield, 1979) | in body mass [g] |
| Gastropoda | Limapontiidae | *Alderia*  *modesta*  (Lovén, 1844) | 3 | 5 | 0.60 | (Seelemann, 1967) |  |
| Gastropoda | Limapontiidae | *Olea*  *hansineensis*  Agersborg, 1923 | 4.3-5.7 | 8 | 0.54-0.71 | (Chia & Skeel, 1973) | Sizes for animals in nature |
| Bivalvia | Mytilidae | *Idas*  *argenteus*  Jeffreys, 1876 | 1.25 | 7 | 0.18 | (Dean, 1993) | Deep water |
| Bivalvia | Mytilidae | *Lithophaga*  *lithophaga*  (Linnaeus, 1758) | 27 | 90 | 0.30 | (Galinou-Mitsoudi & Sinis, 1994) |  |
| Bivalvia | Mytilidae | *Modiolus*  *modiolus*  (Linnaeus, 1758) | 40-50 | 65 | 0.62-0.77 | (Seed & Brown, 1977) |  |
| Bivalvia | Mytilidae | *Mytilus*  *californianus*  Conrad, 1837 | 35-40 | 200 | 0.18-0.20 | (Suchanek, 1981) |  |
| Bivalvia | Mytilidae | *Mytilus*  *edulis*  Linnaeus, 1758 | 6-8 | 70 | 0.09-0.11 | (Brown et al., 1976) |  |
| Bivalvia | Mytilidae | *Mytilus*  *trossulus*  Gould, 1850 | 15-20 | 50 | 0.30-0.40 | (Suchanek, 1981) | As *M. edulis* |
| Bivalvia | Ostreidae | *Crassostrea*  *virginica*  (Gmelin, 1791) | NA | NA | NA | NA | Galtsoff 1974 did not apparently report on age or size at maturity |
| Bivalvia | Pectinidae | *Chlamys*  *islandica*  (O. F. Müller, 1776) | 30-55 | 110 | 0.27-0.50 | (Pedersen, 1994) |  |
| Bivalvia | Arcticidae | *Arctica*  *islandica*  (Linnaeus, 1767) | 12.5-13.1 | 100 | 0.13 | (Rowell et al., 1990) |  |
| Bivalvia | Cardiidae | *Cerastoderma*  *edule*  (Linnaeus, 1758) | 15-20 | 35 | 0.43-0.57 | (Brown et al., 1976) |  |
| Bivalvia | Lasaeidae | *Lasaea*  *subviridis*  Dall, 1899 | NA | NA | NA | NA | Size not provided, only age (Strathmann, 1987) |
| Bivalvia | Vesicomyidae | *Calyptogena*  *magnifica*  Boss & R.D. Turner, 1980 | 120-140 | 240 | 0.40 | (Kennish & Lutz, 1992) | Deep water, ratio reported explicitly in paper |
| Bivalvia | Mactridae | *Spisula*  *solidissima*  (Dillwyn, 1817) | 5-95 | 226 | 0.02-0.42 | (Cargnelli et al., 1999) |  |
| Bivalvia | Mactridae | *Tresus*  *nuttallii*  (Conrad, 1837) | 68 | 202 | 0.34 | (Campbell et al., 1990) |  |
| Bivalvia | Mactridae | *Tresus*  *capax*  (Gould, 1850) | 70 | 149 | 0.47 | (Bourne & Smith, 1972) |  |
| Bivalvia | Tellinidae | *Nitidotellina*  *hokkaidoensis*  (Habe, 1961) | 14-18 | 24.72 | 0.57-0.73 | (Kawai et al., 1993) | as *N. nitidula* |
| Bivalvia | Semelidae | *Abra*  *tenuis*  (Montagu, 1803) | 4 | 6.5 | 0.62 | (Bachelet, 1989) |  |
| Bivalvia | Veneridae | *Dosinia*  *japonica*  (Reeve, 1850) | 47.6 | 72.1 | 0.66 | (Sato, 1994) | as *Phacosoma* |
| Bivalvia | Veneridae | *Dosinia*  *japonica*  (Reeve, 1850) | 38.5 | 64.9 | 0.59 | (Sato, 1994) | as *Phacosoma* |
| Bivalvia | Veneridae | *Dosinia*  *japonica*  (Reeve, 1850) | 35 | 57.5 | 0.61 | (Sato, 1994) | as *Phacosoma* |
| Bivalvia | Veneridae | *Dosinia*  *japonica*  (Reeve, 1850) | 29.8 | 49.5 | 0.60 | (Sato, 1994) | as *Phacosoma* |
| Bivalvia | Veneridae | *Dosinia*  *japonica*  (Reeve, 1850) | 40.8 | 65.3 | 0.62 | (Sato, 1994) | as *Phacosoma* |
| Bivalvia | Veneridae | *Dosinia*  *japonica*  (Reeve, 1850) | 27 | 46.1 | 0.59 | (Sato, 1994) | as *Phacosoma* |
| Bivalvia | Veneridae | *Ruditapes*  *philippinarum*  (Adams & Reeve, 1850) | 15-20 | 65 | 0.23-0.31 | (Holland & Chew, 1974) | As *Venerupis japonica* |
| Bivalvia | Teredinidae | *Bankia*  *setacea*  (Tryon, 1863) | 220 | 520 | 0.42 | (Quayle, 1959) |  |
| Bivalvia | Hiatellidae | *Panopea*  *generosa*  Gould, 1850 | 75 | 197 | 0.38 | (Andersen, 1971) |  |

# Additional results

## Biodiversity loss per taxonomic group

We quantified the ratio between current and historic richness in the shallow subtidal for major taxonomic groups within molluscan classes (Table S7). The systematics was drawn from the World Register of Marine Species.

Table S7. Ratio between current and historical richness on the Mediterranean Israeli shelf for major high-level molluscan taxa.

|  |  | **Gastropoda** | | | | **Bivalvia** | |
| --- | --- | --- | --- | --- | --- | --- | --- |
|  |  | **Vetigastropoda** | **Caenogastropoda** | **Neogastropoda** | **Heterobranchia** | **Pteriomorpha** | **Heterodonta** |
| **Shallow soft**  **(10-40 m)** | Native | 0 | 0.19 | 0.05 | 0.14 | 0.44 | 0.17 |
|  | NIS | 0.33 | 0.80 | No species | 0.90 | 0.40 | 0.58 |
| **Shallow hard**  **(12-25 m)** | Native | 0.09 | 0.37 | 0.10 | 0.22 | 0.07 | 0.31 |
|  | NIS | 0.50 | 1.21 | 1.00 | 1.00 | 0.86 | 0.22 |

# References

Abbott, R. T. (1974). *American seashells. 2nd edition.* Van Nostrand Reinhold.

Andersen, A. M. (1971). *Spawning, growth, and spatial distribution of the geoduck clam,* Panope generosa *Gould, in Hood Canal, Washington.* [University of Washington]. https://digital.lib.washington.edu/researchworks/bitstream/handle/1773/5312/7207315.pdf?sequence=1&isAllowed=y

Appeldoorn, R. S. (1990). Growth of juvenile queen conch, *Strombus gigas* Linnaeus, 1758 off La Parguera, Puerto Rico. *Journal of Shellfish Research*, *9*(1), 59–62.

Bachelet, G. (1989). Recruitment in *Abra tenuis* (Montagu) (Bivalvia, Semelidae), a species with direct development and a protracted meiobenthic phase. In J. S. Ryland & P. A. Tyler (Eds.), *Reproduction, genetics and distributions of marine organisms. 23rd European Marine Biology Symposium, School of Biological Sciences, University of Wales, Swansea, 5-9 September 1988* (pp. 23–30).

Barash, A., & Danin, Z. (1992). *Annotated list of Mediterranean molluscs of Israel and Sinai*. The Israel Academy of Sciences and Humanities.

Berry, A. J. (1989). Factors implicated in the timing of breeding in two contrasted annual intertidal gastropods, *Retusa obtusa* (Montagu) and *Umbonium vestiarium*. In J. S. Ryland & P. A. Tyler (Eds.), *Reproduction, genetics and distributions of marine organisms. 23rd European Marine Biology Symposium, School of Biological Sciences, University of Wales, Swansea, 5-9 September 1988* (pp. 31–36).

Black, R., & Johnson, M. S. (1994). Growth rates in outbreak populations of the corallivorous gastropod *Drupella cornus* (Röding 1798) at Ningaloo Reef, Western Australia. *Coral Reefs*, *13*(3), 145–150. https://doi.org/10.1007/BF00301190

Black, Robert, Turner, S. J., & Johnson, M. S. (1994). The early life history of *Bembicium vittatum* Philippi, 1846 (Gastropoda: Littorinidae). *The Veliger*, *37*(4), 393–399.

Boulding, E. G., & Van Alstyne, K. L. (1993). Mechanisms of differential survival and growth of two species of *Littorina* on wave-exposed and on protected shores. *Journal of Experimental Marine Biology and Ecology*, *169*(2), 139–166. https://doi.org/10.1016/0022-0981(93)90191-P

Bourne, N., & Smith, D. W. (1972). Breeding and growth of the horse clam *Tresus capax* (Gould), in southern British Columbia. *Proceedings of the National Shellfisheries Association*, *62*, 38–46.

Bright, J., Kaufman, D. S., Whitacre, K. E., Ebert, C., Southon, J. R., Albano, P. G., Flores, C., Frazer, T. K., Hua, Q., Kosnik, M. A., Kowalewski, M., Martinelli, J. C., Oakley, D., Parker, W. G., Retelle, M., Ritter, M. D. N., Rivadeneira, M. M., Scarponi, D., Yanes, Y., & Zuschin, M. (submitted). Comparing rapid and standard ^14^C ages from an assortment of biogenic carbonates. *Radiocarbon*.

Bronk Ramsey, C. (2009). Bayesian analysis of radiocarbon dates. *Radiocarbon*, *51*(1), 337–360. https://doi.org/10.1017/S0033822200033865

Brown, R. A., Seed, R., & O’Connor, R. J. (1976). A comparison of relative growth in *Cerastoderma* (=*Cardium*) *edule*, *Modiolus modiolus*, and *Mytilus edulis* (Mollusca: Bivalvia). *Journal of Zoology*, *179*(3), 297–315. https://doi.org/10.1111/j.1469-7998.1976.tb02298.x

Bush, S. L., Santos, G. M., Xu, X., Southon, J. R., Thiagarajan, N., Hines, S. K., & Adkins, J. F. (2013). Simple, rapid, and cost effective: a screening method for ^14^C analysis of small carbonate samples. *Radiocarbon*, *55*(2), 631–640. https://doi.org/10.1017/S0033822200057787

Campbell, A., Bourne, N., & Carolsfeld, W. (1990). Growth and size at maturity of the Pacific gaper *Tresus nuttallii* (Conrad, 1837) in southern British Columbia. *Journal of Shellfish Research*, *9*(2), 273–278.

Cargnelli, L. M., Griesbach, S. J., Packer, D. B., & Weissberger, E. (1999). *Atlantic surfclam,* Spisula solidissima*, life history and habitat characteristics* (NMFS-NE-142; NOAA Technical Memorandum).

Castagna, M., & Kraeuter, J. N. (1994). Age, growth rate, sexual dimorphism and fecundity of knobbed whelk *Busycon carica* (Gmelin, 1791) in a western mid-Atlantic lagoon system, Virginia. *Journal of Shellfish Research*, *13*(2), 581–585.

Chia, F.-S., & Skeel, M. (1973). The effect of food consumption on growth, fecundity, and mortality in a Sacoglossan Opisthobranch, *Olea hansineensis*. *The Veliger*, *16*(2), 153–158.

Choat, J. H., & Black, R. (1979). Life histories of limpets and the limpet-laminarian relationship. *Journal of Experimental Marine Biology and Ecology*, *41*(1), 25–50. https://doi.org/10.1016/0022-0981(79)90079-0

Crofts, D. R. (1937). V - The development of *Haliotis tuberculata*, with special reference to organogenesis during torsion. *Philosophical Transactions of the Royal Society B: Biological Sciences*, *228*(552), 219–268.

Crothers, J. H. (1985). Dog-whelks: an introduction to the biology of *Nucella lapillus* (L.). *Field Studies*, *6*, 291–360.

Dean, H. K. (1993). A population study of the bivalve *Idas argenteus* Jeffreys, 1876, (Bivalvia: Mytilidae) recovered from a submerged wood block in the deep north Atlantic Ocean. *Malacologia*, *35*(1), 21–41.

Eernisse, D. J. (1986). The genus *Lepidochitona* Gray, 1821 Mollusca: Polyplacophora) in the Northeastern Pacific Ocean (Oregonian and Californian provinces). *Zoologische Verhandelingen*, *228*, 3–52.

Fretter, V., & Graham, A. (1976). The prosobranch molluscs of Britain and Denmark. Part 1 – Pleurotomariacea, Fissurellacea and Patellacea. *Journal of Molluscan Studies*, *Suppl. 1*, 1–37.

Fretter, V., & Graham, A. (1985). The prosobranch molluscs of Britain and Denmark. Part 8 – Neogastropoda. *Journal of Molluscan Studies*, *Suppl. 15*, 435–556.

Galinou-Mitsoudi, S., & Sinis, A. I. (1994). Reproductive cycle and fecundity of the date mussel *Lithophaga lithophaga* (Bivalvia: Mytilidae). *Journal of Molluscan Studies*, *60*(4), 371–385. https://doi.org/10.1093/mollus/60.4.371

Gendron, L. (1992). Determination of the size at sexual maturity of the waved whelk *Buccinum undatum* Linnaeus, 1758, in the Gulf of St. Lawrence, as a basis for the establishment of a minimum catchable size. *Journal of Shellfish Research*, *11*(1), 1–7.

Gosselin, L. A., & Qian, P.-Y. (1997). Juvenile mortality in benthic marine invertebrates. *Marine Ecology Progress Series*, *146*, 265–282. https://doi.org/10.3354/meps146265

Heath, H. (1905). The breeding habits of chitons of the Californian coast. *Zoologischer Anzeiger*, *29*(12), 390–393.

Holland, D. A., & Chew, K. K. (1974). Reproductive cycle of the Manila clam (*Venerupis japonica*) from Hood Canal, Washington. *Proceedings of the National Shellfisheries Association*, *64*, 53–58.

Hughes, R. N., & Roberts, D. J. (1980). Reproductive effort of winkles (*Littorina* spp.) with contrasted methods of reproduction. *Oecologia*, *47*(1), 130–136. https://doi.org/10.1007/BF00541788

Kawai, K., Goshima, S., & Nakao, S. (1993). Reproductive cycle and shell growth of the tellin *Nitidotellina nitidula* (Dunker) in Hakodate Bay. *Bulletin of the Faculty of Fisheries - Hokkaido University*, *44*(3), 105–115.

Kemph, S. C., & Willows, A. O. D. (1977). Laboratory culture of the Nudibranch *Tritonia diomedea* Bergh (Tritoniidae: Opisthobranchia) and some aspects of its behavioral development. *Journal of Experimental Marine Biology and Ecology*, *30*(3), 261–276. https://doi.org/10.1016/0022-0981(77)90035-1

Kennish, M. J., & Lutz, R. A. (1992). The hydrothermal vent clam, *Calyptogena magnifica* (Boss and Turner. 1980): a review of existing literature. *Reviews in Aquatic Sciences*, *6*(1), 29–66.

Noda, T., Nakao, S., & Goshima, S. (1995). Life history of the temperate subtidal gastropod *Umbonium costatum*. *Marine Biology*, *122*(1), 73–78. https://doi.org/10.1007/BF00349279

Otway, N. M. (1994). Population ecology of the low-shore chitons *Onithochiton quercinus* and *Plaxiphora albida*. *Marine Biology*, *121*(1), 105–116. https://doi.org/10.1007/BF00349479

Pedersen, S. A. (1994). Population parameters of the iceland scallop (*Chlamys islandica* (Müller)) from West Greenland. *Journal of Northwest Atlantic Fishery Science*, *16*, 75–87.

Poore, G. C. B. (1973). Ecology of New Zealand abalones, *Haliotis* species (Mollusca: Gastropoda). *New Zealand Journal of Marine and Freshwater Research*, *7*(1–2), 67–84. https://doi.org/10.1080/00288330.1973.9515456

Quayle, D. B. (1959). The growth rate of *Bankia setacea* Tryon. In D. L. Ray (Ed.), *Marine boring and fouling organisms* (pp. 175–184). University of Washington Press.

Reimer, P. J., Bard, E., Bayliss, A., Beck, J. W., Blackwell, P. G., Ramsey, C. B., Buck, C. E., Cheng, H., Edwards, R. L., Friedrich, M., Grootes, P. M., Guilderson, T. P., Haflidason, H., Hajdas, I., Hatté, C., Heaton, T. J., Hoffmann, D. L., Hogg, A. G., Hughen, K. A., … Plicht, J. van der. (2013). IntCal13 and Marine13 radiocarbon age calibration curves 0–50,000 years cal BP. *Radiocarbon*, *55*(4), 1869–1887. https://doi.org/10.2458/azu_js_rc.55.16947

Robilliard, G. A. (1971). Range extensions of some Northeast Pacific nudibranchs (Mollusca: Gastropoda: Opisthobranchia) to Washington and British Columbia, with notes on their biology. *The Veliger*, *14*(2), 162–165.

Rowell, T. W., Chaisson, D. R., & McLane, J. T. (1990). Size and age of sexual maturity and annual gametogenic cycle in the ocean quahog, *Arctica islandica* (Linnaeus, 1767), from coastal waters in Nova Scotia, Canada. *Journal of Shellfish Research*, *9*(1), 195–203.

Sakai, S. (1962). Ecological studies on the abalone, *Haliotis discus hannai* Ino–IV. Studies on the growth. *Bulletin of the Japanese Society of Scientific Fishe*, *28*(9), 899–904.

Sato, S. (1994). Analysis of the relationship between growth and sexual maturation in *Phacosoma japonicum* (Bivalvia: Veneridae). *Marine Biology*, *118*(4), 663–672. https://doi.org/10.1007/BF00347514

Seager, J. R. (1982). Population dynamics of the antarctic opisthobranch *Philine gibba* Strebel. *Journal of Experimental Marine Biology and Ecology*, *60*(2), 163–179. https://doi.org/10.1016/0022-0981(82)90156-3

Seed, R., & Brown, R. A. (1977). A comparison of the reproductive cycles of *Modiolus modiolus* (L.), *Cerastoderma* (=*Cardium*) *edule* (L.), and *Mytilus edulis* L. in Strangford Lough, Northern Ireland. *Oecologia*, *30*(2), 173–188. https://doi.org/10.1007/BF00345419

Seelemann, U. (1967). Rearing experiments on the amphibian slug *Alderia modesta*. *Helgoländer Wissenschaftliche Meeresuntersuchungen*, *15*(1), 128–134. https://doi.org/10.1007/BF01618615

Shepherd, S. A. (1986). Studies on Southern Australian abalone (genus *Haliotis*). *Marine Biology*, *90*(2), 231–236. https://doi.org/10.1007/BF00569132

Smith, D. a. S. (1973). The population biology of *Lacuna pallidula* (Da Costa) and *Lacuna vincta* (Montagu) in North-East England. *Journal of the Marine Biological Association of the United Kingdom*, *53*(3), 493–520. https://doi.org/10.1017/S0025315400058720

Spight, T. M. (1975). On a snail’s chances of becoming a year old. *Oikos*, *26*(1), 9. https://doi.org/10.2307/3543270

Spight, Tom M. (1982). Population sizes of two marine snails with a changing food supply. *Journal of Experimental Marine Biology and Ecology*, *57*(2), 195–217. https://doi.org/10.1016/0022-0981(82)90192-7

Strathmann, M. F. (1987). *Reproduction and Development of Marine Invertebrates of the Northern Pacific Coast*. University of Washington Press.

Suchanek, T. H. (1981). The role of disturbance in the evolution of life history strategies in the intertidal mussels *Mytilus edulis* and *Mytilus californianus*. *Oecologia*, *50*(2), 143–152. https://doi.org/10.1007/BF00348028

Switzer-Dunlap, M., & Hadfield, M. G. (1979). Reproductive patterns of Hawaiian Aplysiid gastropods. In S. E. Stancyk (Ed.), *Reproductive ecology of marine invertebrates* (pp. 199–210). University of South Carolina Press.

Underwood, A. J. (1975). Comparative studies on the biology of *Nerita atramentosa* Reeve, *Bembicium nanum* (Lamarck) and *Cellana tramoserica* (Sowerby) (Gastropoda: Prosobranchia) in S.E. Australia. *Journal of Experimental Marine Biology and Ecology*, *18*(2), 153–172. https://doi.org/10.1016/0022-0981(75)90071-4

Yonge, C. M. (1962). On the biology of the mesogastropod *Trichotropis cancellata* Hinds, a benthic indicator species. *The Biological Bulletin*, *122*(1), 160–181. https://doi.org/10.2307/1539329
